# Indobufen *versus* aspirin in dual antiplatelet therapy: a retrospective study of effects on serum uric acid levels and gout attacks in patients post-coronary stenting

**DOI:** 10.7717/peerj.21020

**Published:** 2026-03-27

**Authors:** Sijie Jiang, Lilong Lin, Xiaolin Duan, Jianqin Zhang, Yuqing Hou, Lan Gong, Qingchun Zeng, Hua Zheng

**Affiliations:** 1 Department of Cardiology, Nanfang Hospital, Southern Medical University, Guangzhou, China; 2 State Key Laboratory of Organ Failure Research, Department of Cardiology, Nanfang Hospital, Southern Medical University, Guangzhou, China; 3 UNSW Microbiome Research Centre, St George and Sutherland Clinical Campus, University of New South Wales, Sydney, Australia; 4 School of Medicine, Huaqiao University, Quanzhou, China

**Keywords:** Dual antiplatelet therapy, Indobufen, Serum uric acid, Gout

## Abstract

**Objective:**

This retrospective study compared the effects of indobufen versus aspirin, each combined with clopidogrel, on serum uric acid (SUA) levels and gout attacks in patients undergoing coronary stenting.

**Methods:**

Among 213 enrolled patients, 156 were included after propensity score matching and assigned to either the indobufen plus clopidogrel group (*n* = 78) or the aspirin plus clopidogrel group (*n* = 78). SUA levels were measured at baseline and at the 1-month, 6-month, and 1-year follow-ups. Gout attacks and major cardiovascular events were recorded throughout the study.

**Results:**

Baseline characteristics were comparable. The indobufen group showed significant reductions in SUA levels at all follow-ups (all, *P* < 0.001), whereas levels in the aspirin group remained stable. Although the initial gout attack rates at baseline were similar between groups, the probability of recurrence during follow-up was lower in the indobufen group. Cardiovascular safety profiles were comparable between groups.

**Conclusion:**

Indobufen plus clopidogrel significantly reduced SUA levels and showed a favorable trend in reducing gout recurrences without compromising cardiovascular safety.

## Introduction

Cardiovascular diseases (CVDs) represent a leading cause of morbidity and mortality globally and in China (Writing Group of the Report on Cardiovascular health Diseases in China, 2022; Wal et al., 2024; Wang et al., 2023). For patients with coronary heart disease (CHD) undergoing percutaneous coronary intervention (PCI), dual antiplatelet therapy (DAPT) with aspirin and a P2Y12 inhibitor is a cornerstone of secondary prevention (Thompson & Verheugt, 2014). However, long-term aspirin use is associated with well-documented risks, including gastrointestinal injury and bleeding. Emerging evidence also suggests that even low-dose aspirin may contribute to uric acid retention and hyperuricemia (HUA), thereby increasing the risk of recurrent gout flares. Importantly, the risk of gout attacks appears to be inversely correlated with aspirin dosage. Despite these findings, such adverse effects remain underrecognized in clinical practice (Zhang et al., 2014).

HUA is defined as a fasting serum UA level exceeding 420 µmol/L on two separate occasions, in accordance with the prevailing clinical guideline (Sun et al., 2025). HUA itself is an independent risk factor for CVD and correlates with the severity of vascular lesions in CHD patients. Several studies have suggested that HUA may also contribute to increased all-cause mortality in this population (Lacaita et al., 2025; Wang et al., 2016; Wang et al., 2025).

In light of these concerns, the 2021 expert consensus on managing patients with intolerance or low responsiveness to oral antiplatelet agents (Huo et al., 2021) recommends substituting aspirin with alternative agents such as indobufen or cilostazol in cases of gout occurrence during prolonged DAPT. However, cilostazol use is limited by its potential to increase heart rate and precipitate angina. Indobufen is a selective and reversible cyclooxygenase-1 (COX-1) inhibitor, and offers a favorable safety profile with lower risks of gastrointestinal toxicity and bleeding. The randomized, multicenter, open-label OPTION study (Wu et al., 2023), conducted in China, demonstrated that indobufen-based DAPT significantly reduced bleeding risks without increasing ischemic events within 1 year of stenting. These findings support the viability of indobufen as an alternative to aspirin.

Despite these findings, the comparative effects of indobufen- *versus* aspirin-based DAPT on serum UA (SUA) levels and gout incidence remain unclear. Therefore, this study aims to directly compare the impact of indobufen *versus* aspirin, each combined with clopidogrel, on SUA levels and gout incidence in patients post-coronary stenting. Clarifying these effects may provide valuable insights for optimizing DAPT strategies in patients with elevated UA or a history of gout.

## Materials and Methods

### Research subjects

This study included patients who underwent coronary stent implantation at Nanfang Hospital of Southern Medical University, China, between January 2020 and December 2020. The inclusion criteria for the observation group where: (1) age ≥ 18 years, post-coronary intervention, and on regular DAPT; (2) previously used aspirin combined with clopidogrel and then adjusted to indobufen (0.1 g 2/day) combined with clopidogrel (75 mg 1/day) due to a history of intolerance events, such as resolved or stably managed digestive bleeding, gastrointestinal ulcers, recent gout attack, or elevated SUA levels (SUA ≥ 360 µmol/L; above the recommended treatment target for HUA patients with cardiovascular comorbidities Sun et al., 2025); and (3) a history of gastrointestinal ulcers or bleeding; thus, ineligible for aspirin use. The inclusion criteria for the control group were: (1) age ≥ 18 years, post coronary intervention and regularly taking dual antiplatelet drugs; and (2) baseline SUA level ≥ 360 µmol/L, but the DAPT regimen of aspirin (0.1 g/day) combined with clopidogrel (75 mg/day) was adopted. The exclusion criteria were: (1) severe heart failure (NYHA grade III and above); (2) acute ST-segment elevation myocardial infarction (MI); (3) incomplete revascularization of multiple coronary artery lesions or severe complications of coronary interventional surgery; (4) severe infection; (5) severe liver function impairment (alanine aminotransferase (ALT) or aspartate aminotransferase (AST) levels ≥ 1.5 times the upper limits of the normal values), active hepatitis, or cirrhosis; (6) severe renal impairment (serum creatinine (SCR) level ≥ 1.5 times the upper limit of the normal value) in patients who had received or were receiving kidney transplantation, hemodialysis, or peritoneal dialysis; (7) abnormal thyroid function; (8) abnormal adrenal function; (9) autoimmune diseases; (10) systemic blood diseases; (11) malignant tumors; (12) allergy to aspirin, clopidogrel, or other antiplatelet drugs; (13) thrombocytopenia (platelet (PLT) count of <100 ×10^9^/L), anemia (hemoglobin (HGB) level of <90 g/L), active bleeding, or any clinically significant bleeding event within 3 months prior to enrollment, or significant tendency of bleeding; (14) intractable gout attacks and tophus formation; and (15) long-term use of drugs that affect UA metabolism, including uric acid-lowering drugs (*e.g.*, allopurinol, febuxostat, and benzbromarone), diuretics (*e.g.*, furosemide and hydrochlorothiazide), antituberculosis drugs (*e.g.*, pyrazinamide and ethambutol), immunosuppressants (*e.g.*, tacrolimus and cyclosporine), colchicine, losartan and niacin. Sodium bicarbonate is commonly used to alkalize urine, relieve pain, and treat other symptoms and does not affect the level of UA therefore, it was not excluded.

This was a retrospective, observational study that did not involve prospective intervention. The Medical Ethics Committee of Nanfang Hospital of Southern Medical University approved the study protocol and waived the requirement for informed consent (protocol code #NFEC-2022-312; date of approval: 12 August 2022).

### Data collection

The hospital medical record system was used to collect relevant patient data including age, sex, medical history (hypertension, type 2 diabetes, CHD, gout, and relevant medications) and the blood-test indexes of patients before treatment and 1-month, 6-month, and 1-year after treatment including routine blood tests (HGB, PLT), lipid parameters (low-density lipoprotein cholesterol (LDL-C)), renal function indicators (SCR and SUA), and liver function indicators (ALT and AST). The incidences of gout, serious gastrointestinal adverse events (*e.g.*, digestive ulcers and digestive bleeding), and major cardiovascular adverse events (MACE) (*e.g.*, refractory angina pectoris, new heart failure, myocardial infarction, cardiovascular death, and stroke) were also recorded.

### Statistical analysis

Statistical analyses were performed using IBM SPSS Statistics for Windows, version 26.0 (IBM Corp., Armonk, NY, USA). Categorical data were expressed as number (%), and the *χ*^2^ test or Fisher’s exact test was used for comparisons between groups. Normally distributed data were presented as mean ± standard deviation. Paired data before and after treatment were also tested using student’s paired *t*-test while student’s independent *t*-test was used for comparisons between two groups. Non-normally distributed data were presented as median (25% percentile, 75% percentile). The Wilcoxon signed-rank test was used for comparison of paired data before and after treatment, and the Mann–Whitney U test was used for comparisons between the two groups. All tests were 2-sided, and statistical significance was set at *P* < 0.05.

Due to the imbalance of baseline SUA levels between groups, propensity score matching (PSM) was used to select paired cases from the two groups controlling for baseline SUA levels. This new subset data after PSM was applied to all analyses. The matching method used was “nearest”, sample size ratio between groups was set at 1, and the caliper value was 0.5. The statistical software R (version 4.4.0) and the package ‘MatchIt’ were used to perform PSM.

A sample size estimation was performed based on a conservative assumption that the groups after PSM could be compared as independent samples. Given a standardized effect size (*δ*/*σ*) of 0.5 (medium effect), a 2-sided significance level (*α*) of 0.05, and a statistical power (1 − *β*) of 80%, calculation using PASS software (version 15.0) indicated that at least 34 subjects per group were required. As the PSM design effectively controls for confounders and improves comparability, the final matched cohort in this study met and exceeded this estimated sample size, thereby providing adequate power for the primary analysis.

## Results

### Comparison of baseline data between the two groups

A total of 213 patients who underwent coronary stent implantation at Nanfang Hospital of Southern Medical University between January 2020 and December 2020 were included in this study. All patients received DAPT following the procedure. Based on the antiplatelet regimen, patients were allocated to either the observation group (*n* = 83), receiving indobufen combined with clopidogrel, or the control group (*n* = 130), receiving aspirin combined with clopidogrel.

As shown in Table 1, all baseline characteristics were well-balanced between the two groups following PSM, with no statistically significant differences observed in either parametric or non-parametric tests. Specifically, no statistically significant differences were observed in age, sex distribution, comorbidities, or baseline laboratory indicators, HGB, PLT count, ALT, AST, SCR, SUA, estimated glomerular filtration rate (eGFR), and LDL-C. Consequently, all subsequent analyses in this study were conducted using the matched cohort.

**Table 1:** Comparison of baseline data between the two groups after PSM.

Parameters	Observation group (*n* = 78)	Control group (*n* = 78)	*P*-value
**Basic data**			
Age (years)	60.03 ± 11.74	59.44 ± 11.14	0.748
Male (n (%))	59 (75.64%)	60 (76.92%)	0.851
BMI	24.40 (22.50, 27)	24.64 (22.83, 27.78)	0.326
**Past medical history**			
Hypertension (n (%))	62 (79.49%)	59 (75.64%)	0.565
Diabetes (n (%))	40 (51.28%)	33 (42.31%)	0.261
**Serum indicators**			
SUA (μ mol/L)	426 (377.50, 472.75)	425.50 (377.50, 473)	0.714
HGB (g/L)	138.82 ± 20.55	138.96 ± 18.21	0.964
LDL-C (mmol/L)	2.61 ± 1.12	2.95 ± 1.09	0.057
ALT (U/L)	24.50 (15, 35)	21.50 (16, 31)	0.840
AST (U/L)	22 (16, 27)	20.50 (17, 27)	0.808
SCR (μ mol/L)	86.50 (72, 101)	91 (79, 98.25)	0.245
eGFR	79.02 (67.11, 91.41)	75.33 (65.57, 87.07)	0.408
**History of drug therapy**			
Statins (n (%))	73 (93.59%)	76 (97.44%)	0.442
Atorvastatin (n (%))	51 (69.86%)	55 (72.37%)	0.736
Beta blockers (n (%))	44 (56.41%)	47 (60.26%)	0.626
ACEI/ARB (n (%))	48 (61.54%)	49 (62.82%)	0.869
CCB (n (%))	26 (33.33%)	22 (28.21%)	0.488

**Notes.**

ACEI Angiotensin converting enzyme inhibitor ALT alanine aminotransferase AST aspartate aminotransferase ARB angiotensin receptor blockers BMI Body Mass Index CCB calcium channel blocker HGB hemoglobin LDL-C low-density lipoprotein cholesterol SCR serum creatinine SUA serum uric acid eGFR estimated glomerular filtration rate

### Comparison of SUA levels between the two groups during follow-up

SUA levels after treatment were compared between the observation group (*n* = 78) and the control group (*n* = 78). As presented in Table 2, at 1 month, the mean SUA level was significantly lower in the observation group compared to the control group (377.53 ± 74.32 µmol/L *vs.* 426.16 ± 78.12 µmol/L, *P* ≤ 0.01). Similarly, at 6 months, the observation group maintained a lower mean SUA level relative to the control group (377.76 ± 68.22 µmol/L *vs.* 413.44 ± 60.72 µmol/L, *P* ≤ 0.01). This trend persisted at the 1-year follow-up, with SUA levels remaining significantly lower in the observation group than in the control group (381.22 ± 73.74 µmol/L *vs.* 422.71 ± 64.24 µmol/L, *P* ≤ 0.01). These findings indicate that patients receiving indobufen combined with clopidogrel consistently exhibited lower SUA levels throughout the follow-up period compared with those receiving aspirin combined with clopidogrel.

**Table 2:** Comparison of SUA levels between the two groups during follow-up.

Parameters	Observation group (*n* = 78)	Control group (*n* = 78)	*P*-value
SUA (μ mol/L)			
Baseline	426 (377.50, 472.75)	425.50 (377.50, 473)	0.714
1-month	377.53 ± 74.32^a^	426.16 ± 78.12	<0.001^*^
6-month	377.76 ± 68.22^a^	413.44 ± 60.72	0.002^*^
1-year	381.22 ± 73.74^a^	422.71 ± 64.24	<0.001^*^
SUA change amount (compared to baseline)			
1-month	−56.79 ± 68.68	−6.01 ± 74.30	<0.001^*^
6-month	−68.31 ± 86.98	−25.98 ± 81.42	0.004^*^
1-year	−57.14 ± 72.67	−7.63 ± 70.17	<0.001^*^
SUA change ratio % (compared to baseline)			
1-month	−12.62 ± 14.07	−0.68 ± 16.55	<0.001^*^
6-month	−14.02 ± 16.59	−4.41 ± 16.81	0.001^*^
1-year	−12.39 ± 14.66	−0.86 ± 15.53	<0.001^*^

**Notes.**

SUA, Serum uric acid.

* Indicates statistically significant difference.

a Compared to its own baseline, *P* ≤ 0.05.

We further analyzed the longitudinal changes in SUA levels within each group (Table 2). In the observation group, SUA levels decreased by an average of 12.62%, 14.02%, and 12.39% at the 1-month, 6-month, and 1-year follow-ups, respectively, compared with baseline. Both the absolute changes and percentage reductions in SUA levels were statistically significant at all time points (all, *P* ≤ 0.01, denoted by superscript ‘^a^’ in Table 2). In contrast, in the control group, SUA levels decreased marginally by an average of 0.68%, 4.41%, and 0.86% at the corresponding time points. However, these reductions were not statistically significant when compared to baseline (all, *P* > 0.05).

**Figure 1:**
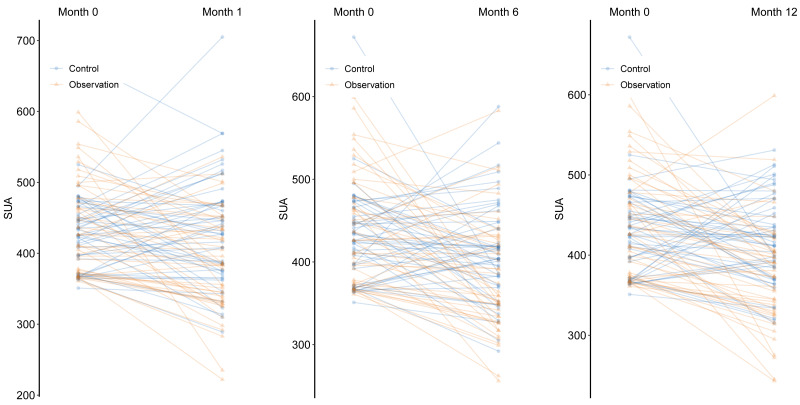
Comparison of SUA (serum uric acid) levels (μ mol/L) between the observation group and the control group at 1-month, 6-month and 1-year post-treatment comparing to baseline using the slope plot of all individuals.

Notably, several patients in the observation group had initially received aspirin-based DAPT but were subsequently switched to indobufen due to adverse events such as gastrointestinal bleeding, gastrointestinal ulcers, recent gout flares, or elevated SUA levels. In these switched patients, the median duration of prior aspirin therapy was 66 days (range: 7–279 days). These findings further suggest that, compared with aspirin, indobufen therapy is associated with a greater tendency to reduce SUA levels (Table 2 and Fig. 1). As illustrated in Fig. 1, the slope plots demonstrate a significant downward trend in SUA levels over time in the observation group, whereas SUA levels in the control group remained largely unchanged throughout the follow-up period.

### Comparison of the proportions of gout attacks between the two groups

**Figure 2:**
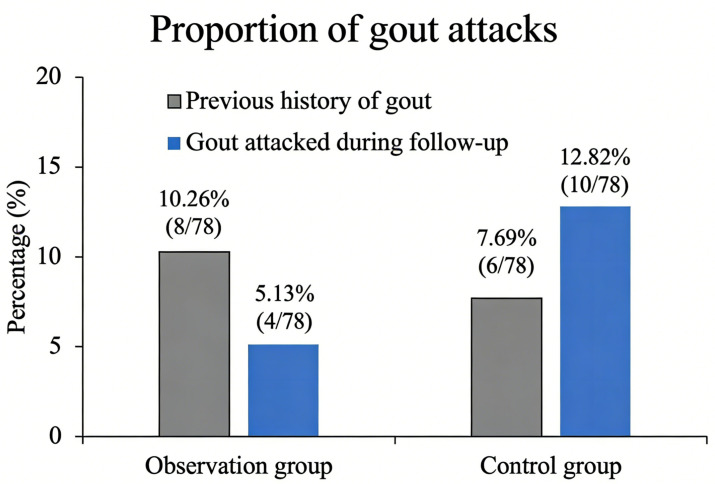
Comparison of the proportions of gout attacks (%) before and after treatment in the observation group and the control group.

We subsequently compared the incidence of gout attacks between the two groups (Table 3 and Fig. 2). In the observation group, eight patients (10.26%) had a prior history of gout, and among them, four patients (5.13%) experienced gout attacks during the follow-up period. In the control group, six patients (7.69%) had a history of gout; notably, a total of 10 patients (12.82%) experienced gout attacks during follow-up, including all six patients with pre-existing gout. Across both groups, the duration of prior gout history ranged from 1 month to 5 years, and no patients exhibited tophus (gout stone) formation at baseline or during follow-up. Statistical analysis revealed no significant differences between the two groups in the prevalence of prior gout history or the incidence of gout attacks during the follow-up period (both, *P* > 0.05; Table 3 and Fig. 2).

Patients in the observation group had previously exhibited intolerance to their initial DAPT regimen for various reasons, prompting a change in antiplatelet therapy. Given that gout attacks represent an important manifestation of aspirin intolerance, the proportion of patients with a history of gout in the observation group was initially higher than that in the control group, prior to adjustment for the current DAPT regimen. Among patients receiving aspirin combined with clopidogrel, the incidence of gout attacks significantly increased during the follow-up period. In contrast, no new cases of gouty arthritis were observed in the observation group throughout follow-up, and the incidence of gout attacks was notably reduced. These findings suggest that the DAPT regimen of indobufen combined with clopidogrel was more effective in lowering the incidence of gout attacks compared with the aspirin-based regimen.

### Comparison of the safety of the 2 DAPT regimens

Routine laboratory parameters, including complete blood counts, lipid profiles, liver function tests, renal function indicators, and other clinical markers, were assessed in both groups after 1 year of treatment with the current DAPT regimen (Table 4). No statistically significant differences were observed between the 2 groups across any of these parameters (all, *P* > 0.05).

**Table 3:** Comparison of the proportion of gout attacks between the two groups.

Parameters	Observation group (*n* = 78)	Control group (*n* = 78)	*P*-value
Previous history of gout (n (%))	8 (10.26%)	6 (7.69%)	0.575
Gout attacked during follow-up (n (%))	4 (5.13%)	10 (12.82%)	0.093

**Table 4:** Comparison of safety indexes between the two groups during 1- year follow-up.

Parameters	Observation group (*n* = 78)	Control group (*n* = 78)	*P*-value
HGB (g/L)	140.02 ± 20.23	140.35 ± 15.15	0.916
LDL-C (mmol/L)	1.62 (1.34, 2.20)	1.73 (1.28, 2.23)	0.812
ALT (U/L)	21 (14, 32)	21 (18, 28)	0.891
AST (U/L)	20 (17, 25)	20 (17, 24.75)	0.777
SCR (μ mol/L)	87 (75, 102)	90 (79.25, 103.25)	0.498
eGFR	80.34 ± 24.45	77.63 ± 19.16	0.479
MACE (n (%))	4 (5.13%)	4 (5.13%)	1.000

**Notes.**

HGB hemoglobin LDL-C low-density lipoprotein cholesterol MACE major cardiovascular adverse events SCR serum creatinine

During the follow-up period, major adverse cardiovascular events (MACE) occurred in four patients (5.13%) in the observation group and four patients (5.13%) in the control group, with no significant difference between the groups (*P* > 0.05; Table 4). Among these events, one patient in the observation group and two patients in the control group required repeat stent implantation. The remaining MACE cases in both groups presented with exertional angina pectoris; notably, no cases of recurrent acute MI, cardiac death, or other serious cardiovascular events were reported.

Additionally, one patient in the control group developed melena during follow-up. The fecal occult blood test was positive; however, hemoglobin levels did not decline significantly. After receiving intensified gastroprotective therapy, the patient’s fecal occult blood test returned to negative within 1 week.

### Comparison of SUA levels between subgroups during follow-up

A subgroup analysis was performed based on the clinical cutoff for HUA (420 µmol/L). In the subgroup with baseline SUA < 420 µmol/L, both the observation and control groups comprised 35 patients each (Table 5). The baseline SUA levels were comparable between the two groups, with 382.89 ± 22.28 µmol/L in the observation group and 383.54 ± 20.19 µmol/L in the control group.

**Table 5:** Comparison of SUA levels between subgroup-1 (SUA <420 μ mol/L) during follow-up.

Parameters	Observation group (*n* = 35)	Control group (*n* = 35)	*P*-value
Age (years)	61.06 ± 11.20	58.17 ± 9.68	0.253
Male (n (%))	23 (65.71%)	27 (77.14%)	0.290
Hypertension (n (%))	25 (71.43%)	24 (68.57%)	0.794
Diabetes (n (%))	15 (42.86%)	14 (40.00%)	0.808
SUA (μ mol/L)			
Baseline	382.89 ± 22.28	383.54 ± 20.19	0.898
1-month	332.72 ± 50.27^a^	395.71 ± 61.69	<0.001^*^
6-month	353.96 ± 50.89^a^	395.48 ± 59.24	0.008^*^
1-year	335.48 ± 42.71^a^	405.75 ± 56.06	<0.001^*^
SUA change amount (compared to baseline)			
1-month	−47.50 ± 41.29	14.03 ± 59.73	<0.001^*^
6-month	−30.63 ± 47.67	13.67 ± 60.45	0.004^*^
1-year	−46.03 ± 37.53	20.41 ± 56.69	<0.001^*^
SUA change ratio % (compared to baseline)			
1-month	−12.66 ± 11.11	3.74 ± 15.40	<0.001^*^
6-month	−7.97 ± 12.33	3.76 ± 15.53	0.003^*^
1-year	−12.07 ± 10.08	5.46 ± 15.12	<0.001^*^

**Notes.**

SUA, Serum uric acid.

* Indicates statistically significant difference.

a Compared to its own baseline, *P* ≤ 0.05.

Over the follow-up period, the observation group demonstrated reductions in SUA levels of 12.66%, 7.97%, and 12.07% at the 1-month, 6-month, and 1-year follow-up, respectively. In contrast, the control group exhibited increases in SUA levels of 3.74%, 3.76%, and 5.46% at the corresponding time points. The difference in the degree of SUA change between the 2 subgroups was statistically significant across all follow-up periods (Table 5).

In the subgroup with baseline SUA ≥ 420 µmol/L, both the observation and control groups included 43 patients each (Table 6). The baseline SUA levels were 488.67 ± 60.15 µmol/L in the observation group and 477.74 ± 54.81 µmol/L in the control group. During follow-up, the observation group demonstrated substantial reductions in SUA levels of 12.58%, 18.10%, and 12.67% at the 1-month, 6-month, and 1-year time points, respectively. In comparison, the control group exhibited more modest reductions of 4.19%, 10.07%, and 6.47% at the corresponding time points (Table 6). The observation group, which had a higher baseline SUA level, achieved a greater reduction in SUA compared to the control group, with a statistically significant difference observed at the 6-month follow-up (*P* = 0.037).

**Table 6:** Comparison of SUA levels between subgroup-2 (SUA ≥420 μ mol/L) during follow-up.

Parameters	Observation group (*n* = 43)	Control group (*n* = 43)	*P*-value
Age (years)	59.19 ± 12.22	60.47 ± 12.22	0.629
Male (n (%))	36 (83.72%)	33 (76.74%)	0.417
Hypertension (n (%))	37 (86.05%)	35 (81.40%)	0.559
Diabetes (n (%))	25 (58.14%)	19 (44.19%)	0.196
SUA (μ mol/L)			
Baseline	488.67 ± 60.15	477.74 ± 54.81	0.381
1-month	417.36 ± 69.76^a^	450.36 ± 81.98	0.066
6-month	393.83 ± 74.12^a^	425.87 ± 59.30^a^	0.037^*^
1-year	420.24 ± 72.54^a^	437.78 ± 67.96^a^	0.300
SUA change amount (compared to baseline)			
1-month	−65.06 ± 85.85	−21.95 ± 81.37	0.029^*^
6-month	−93.75 ± 98.19	−53.44 ± 83.35	0.053
1-year	−66.62 ± 92.33	−32.56 ± 72.26	0.089
SUA change ratio % (compared to baseline)			
1-month	−12.58 ± 16.42	−4.19 ± 16.79	0.032^*^
6-month	−18.10 ± 17.94	−10.07 ± 15.43	0.036^*^
1-year	−12.67 ± 17.81	−6.47 ± 13.79	0.107

**Notes.**

SUA, Serum uric acid.

* Indicates statistically significant difference.

a Compared to its own baseline, *P* ≤ 0.05.

## Discussion

Aspirin, a derivative of salicylic acid, has long been known to exert bidirectional effects on UA metabolism. As early as the late 1950s, Leung et al. (2022) reported that salicylic acid inhibits UA excretion at low doses while promoting excretion at higher doses. Similarly, Caspi et al. (2000) demonstrated that low-dose aspirin (75 mg/day) reduced UA excretion by approximately 15%, with this effect diminishing as the aspirin dose increased. The underlying mechanisms have since been linked to the function of UA anion transporter-1 (URAT1). Specifically, at low doses, salicylic acid enhances URAT1-mediated reabsorption of UA, leading to UA accumulation, whereas at higher doses, it inhibits URAT1 activity, thereby promoting UA excretion (Enomoto et al., 2002). In addition, salicylic acid inhibits multidrug resistance-associated protein-4 (MRP4), further reducing UA excretion, although this effect appears to be secondary (Mishra, Harwansh & Mazumder, 2025). Aspirin also exhibits high affinity for organic anion transporters 1(OAT1) and OAT3, which contributes to reduced renal excretion of UA and elevated SUA levels (Khamdang et al., 2002).

Clinical evidence aligns with these mechanistic findings. Zhang et al. (2014) reported that continuous low-dose aspirin use significantly increases the risk of recurrent gout flares. Compared with non-use, daily aspirin intake of ≤ 325 mg elevated the risk of gout attacks by 81%, and doses ≤ 81 mg increased the risk by as much as 91%. A population-based study in the UK further revealed that at least one-third of patients with gout were current or former aspirin users (Kuo et al., 2015).

Nevertheless, conflicting findings have also been reported. Clinical studies by Zhang et al. (2020) and Li, Fan & Liu (2021) indicated that long-term aspirin use in patients with hyperuricemia did not elevate SUA levels, and even showed a slight downward trend. The authors speculated that variations in renal function may explain these discrepancies, though the precise mechanisms remain unclear and require confirmation in large-scale prospective studies. In our study, the control group exhibited only a slight, non-significant upward trend in SUA levels, which contrasts with Caspi’s findings. This discrepancy may reflect our limited sample size or differences in renal function among patients. Further studies are needed to elucidate the relation between aspirin’s impact on UA metabolism and renal function.

Indobufen, a selective COX-1 inhibitor, is widely recognized as an alternative to aspirin. Meta-analyses have demonstrated that indobufen is superior to other antiplatelet agents in reducing adverse events, such as gastrointestinal reactions and bleeding, in patients with acute coronary syndromes and ischemic cardiovascular or cerebrovascular disease (Cui et al., 2022; Yang et al., 2017). To date, however, no clinical study has confirmed a direct UA-lowering effect of indobufen. Compared with aspirin, indobufen possesses a non-salicylic acid structure and lacks activity on key UA transporters such as URAT1, OAT1, and OAT3 in renal tubular epithelial cells. Furthermore, indobufen exerts minimal influence on endothelial COX-1 and prostacyclin (PGI2) synthesis (Lee, Sung & Choi, 2016). Given the critical role of PGI2 in maintaining renal physiology (Cao et al., 2019), indobufen may have a limited impact on glomerular filtration, thereby preserving UA excretion.

Additionally, approximately one-third of SUA elimination occurs *via* the gastrointestinal tract. Recent research has highlighted the role of the intestine in UA homeostasis, particularly the high expression of the ATP-binding cassette transporter G2 (ABCG2) in the gut, which is now considered the principal mediator of intestinal UA excretion (Ichida et al., 2012). Emerging evidence also suggests that the gut microbiota plays a role in UA metabolism. Thus, it is plausible that indobufen may influence UA handling indirectly by modulating ABCG2 expression or altering gut microbiota composition. Nevertheless, these hypotheses require validation through further mechanistic studies.

Several limitations of this study should be acknowledged. First, its retrospective and non-randomized design introduces inherent risks of selection bias and unmeasured confounding, despite our use of PSM to balance key baseline variables. Second, the sample size, though adequate for the primary analysis, may limit the statistical power for subgroup analyses and the detection of rare adverse events. Third, the follow-up duration was limited to 1 year, which precludes assessment of the long-term effects of indobufen on SUA metabolism and cardiovascular outcomes. Finally, our primary endpoints were changes in SUA levels and gout incidence, which are surrogate markers; larger studies with hard clinical endpoints are warranted to confirm the clinical implications of these findings.

An interesting finding was the observed decline in SUA levels within the control group. While this study was not designed to elucidate the causes, potential contributors may include enhanced dietary awareness among patients with elevated baseline SUA or the influence of concomitant medications. Nevertheless, despite this background reduction, the indobufen group demonstrated a significantly greater and more consistent decrease in SUA over time, robustly supporting its UA-lowering efficacy.

## Conclusions and Prospects

This study provides the first direct comparison of the effects of indobufen- *versus* aspirin-based DAPT on SUA levels and gout incidence. The results demonstrate that indobufen combined with clopidogrel significantly reduces SUA levels and the risk of gout attacks compared to the conventional aspirin-clopidogrel regimen, while maintaining a comparable cardiovascular safety profile. These findings suggest that for post-intervention patients with elevated SUA or a history of gout, indobufen represents a viable and preferable alternative antiplatelet strategy, given its beneficial effects on UA metabolism and comparable cardiovascular safety. Further studies with larger sample sizes and longer follow-up are warranted to confirm the long-term efficacy and elucidate the underlying mechanisms.

##  Supplemental Information

10.7717/peerj.21020/supp-1 Supplemental Information 1 Raw data

10.7717/peerj.21020/supp-2 Supplemental Information 2 STROBE checklist

## Additional Information and Declarations

### Data Availability

The following information was supplied regarding data availability: The raw data is available in the Supplemental Files.
